# COVID-19 Exposure, Stress, and Mental Health Outcomes: Results From a Needs Assessment Among Low Income Adults in Central North Carolina

**DOI:** 10.3389/fpsyt.2021.790468

**Published:** 2022-01-20

**Authors:** Andréa R. Kaniuka, Robert J. Cramer, Corrine N. Wilsey, Jennifer Langhinrichsen-Rohling, Annelise Mennicke, Alexandra Patton, Meagan Zarwell, Carmen P. McLean, Yu-Jay Harris, Sharon Sullivan, Glori Gray

**Affiliations:** ^1^Department of Public Health Sciences, University of North Carolina at Charlotte, Charlotte, NC, United States; ^2^Department of Psychological Sciences, University of North Carolina at Charlotte, Charlotte, NC, United States; ^3^School of Social Work, University of North Carolina at Charlotte, Charlotte, NC, United States; ^4^National Center for PTSD, Dissemination and Training Division, VA Palo Alto Health Care System, Menlo Park, CA, United States; ^5^Department of Psychiatry and Behavioral Sciences, Stanford University, Stanford, CA, United States; ^6^Psychology for All, Charlotte, NC, United States

**Keywords:** COVID-19, coping, stress, mental health, suicide

## Abstract

This study focuses on identifying COVID-19 related exposure, stress, and mental health concerns in the larger Charlotte, North Carolina region, an area with many low-income and under resourced communities. A community-academic partnership conducted a regional COVID-19 needs assessment. Low-income adults (*N* = 156) completed an online-administered survey of demographic information, COVID-19 exposure, stress, coping-related factors, and mental health. Frequency data showed that common COVID-19 related stressors included job exposure, lost job/income, and increased home responsibilities. Frequency data further showed elevated screening risk rates for mental health concerns were observed for post-traumatic stress (83.3%), depression (52.2%), problematic drinking (50.0%), generalized anxiety (43.0%), and suicide (40.4%). Bivariate correlation and multivariate regression models identified robust mental health risk factors including COVID-19 related stress affecting close persons, fear/worry reaction to the pandemic, and use of venting as a coping strategy; protective factors included active coping and problem-focused coping beliefs. Findings are discussed with respect to informing regional public health efforts during the pandemic.

In January 2020, the novel coronavirus (COVID-19) was declared a public health emergency in the United States after the World Health Organization reported the spread of the virus as an international problem (1). As of September 2021, an estimated 220 million cases were documented worldwide, including over 650,000 deaths in the United States alone (2). In addition to proximal COVID-19 symptoms and death, the pandemic and associated public health interventions (e.g., home-confinement, social distancing) have resulted in psychosocial and mental health impacts (3). As such, the International COVID-19 Suicide Prevention Research Collaboration released a global call to action anticipating rising adverse mental health effects due to the ongoing pandemic (4). The present study contributes to that call through a focus on vulnerable, low-income adults in an urban region of the southern United States.

## What Do We Know About Stress, Behavioral Responses, and Mental Health in Pandemics?

Globally, rates of mental disorders (e.g., depression, anxiety, post-traumatic stress disorder) are a concern, with a prevalence of approximately 22% (5); further, low-income individuals are at increased risk of mental health disorders and suicide attempts (6). Public health emergencies may exacerbate rates of negative mental health outcomes. Individuals and communities experiencing public health emergencies may endure a number of emotional and behavioral stressors and behavioral impacts, including increased distress or mental health conditions, and increased substance use (7). For example, survey data suggests that quarantine, isolation, and other social distancing measures during pandemic-focused disasters are associated with stress and the risk for post-traumatic stress disorder (8). In Singapore, one study identified increased rates of psychiatric symptoms (e.g., post-traumatic effects) ranging between 22.9 and 25.8% among the general population (9). The long-term mental health outcomes following the 2009 influenza strain H1N1 (i.e., swine flu) pandemic included increased rates of anxiety, depression, and a greater risk for post-traumatic stress disorder (10).

Recent studies similarly indicate that poor mental health outcomes may be associated with the ongoing COVID-19 pandemic (3, 11, 12). Globally, rates of mental health issues, including depression, anxiety, and post-traumatic stress (PTS) increased during the initial months of the COVID-19 pandemic compared to pre-pandemic rates (13, 14). Further, besides PTS, the pandemic has been linked with increased feelings of apprehension, boredom, anger, fear, uncertainty, loneliness, stress, anxiety, and suicide ideation (14–17). For instance, interviews with college students in the United States showed an escalation in rates of stress and anxiety during the pandemic (18). Both the virus and associated safety guidelines represent stressors, with extended periods of social distancing straining mental health (15, 19). For example, findings early on in the pandemic (March 2020), suggested that social distancing was linked with worse mental health (e.g., symptoms of depression, anxiety, insomnia, and acute stress) (20). Other documented stressors associated with the pandemic include financial difficulties, fear of becoming physically ill, and concern about accessing mental health resources (21–24).

Repeated exposure to global media coverage related to the virus and social distancing can also perpetuate stress and anxiety and influence noncompliance of public health directives (e.g., mask mandates), leading to absence of help-seeking behaviors (25). In terms of reactions to the pandemic and its media coverage, research conducted in multiple countries demonstrates adverse mental health impacts including sleep disturbance, attention difficulties, extreme increased substance use, less social interaction, and increased social isolation, as well as lowered perceived physical health (18, 26).

Understanding how these stressors interact with known social risk factors is critical to understanding the mental health impact of the pandemic. Studies of the impact of COVID-19 on mental health have largely focused on samples of convenience or have focused on specific occupational groups (e.g., medical staff) or medically vulnerable populations. However, low socioeconomic status is a critical social risk factor that may heighten the risk for negative mental health outcomes, warranting research with low socioeconomic status populations. For example, longitudinal research indicates that overall depressive symptoms among U.S. adults increased from early in the pandemic compared to 1 year later, with low household income exacerbating depressive symptoms (27). An important aim of the present study is to examine a range of stressors attributed to COVID-19 among individuals who are disproportionately affected by socioeconomic systems that lead to poverty.

Emerging evidence suggests the pandemic may result in both helpful and unhelpful coping strategies (e.g., venting, active coping, substance use) (23, 24). Documented efforts to cope with pandemic-related stress include positive (e.g., social support, mindfulness) and negative (e.g., substance use, social withdrawal) coping techniques (28, 29). For example, healthy (e.g., mindfulness) and unhealthy (e.g., sleeping longer) self-management strategies and seeking social support were common among college students grappling with the pandemic (18). An additional aim of this study is to quantify COVID-19 related exposure and stress, especially as they relate to the mental health and coping of adults with lower incomes in the Southern United States.

## Why Central North Carolina?

Central North Carolina (NC), including Charlotte and its surrounding areas, has a deep history of social and economic inequality. Approximately 14% of the region lives both below the poverty line and without health insurance (30). More than 50% of the region are people of color, with over 30% identifying as African American and another 14% as Hispanic. In addition, at least 15% are immigrants and refugees. Thus, a substantial proportion of the population is exposed to socioeconomic systems that lead to poverty, institutional racism, and discrimination that targets Black, Indigenous People of Color (BIPOC), and socio-political policies that target undocumented immigrants, all of which may heighten the negative impact of the pandemic on mental health and/or poor coping strategies. Indeed, findings show that the COVID-19 pandemic has disproportionately affected members of low-income and urban communities of color (31, 32), likely exacerbated by pre-existing and ongoing social, health, and economic inequities. The demographic diversity of central NC leaves the area particularly vulnerable to long-lasting negative impacts of COVID-19. In NC, more than 31,000 people are estimated to be at risk of complications due to COVID-19, with patterns worse for racially and ethnically minoritized groups and those without health insurance (33). BIPOC individuals are more likely to have pre-existing conditions such as asthma or diabetes, and are more likely to work jobs deemed essential, increasing their likelihood of exposure to SARS-CoV-2, and ultimately leading to higher rates of COVID-19 mortality (34, 35). The demographic, financial, and social composition of central NC raises concern about the long-term impact of the local COVID-19 pandemic.

The broader public health research literature helped us identify starting points for this COVID-19 needs assessment. For instance, a June 2020 CDC study regarding mental health during the pandemic showed that 40% of those surveyed reported struggling with mental health or substance use (36). Specifically, 31% of individuals reported symptoms of anxiety/depression, 26% indicated having a trauma-related concern, 13% started or increased substance use, and 11% considered suicide. Further, substance abuse and suicidal ideation frequencies were higher among young adults and members of racial/ethnic minority groups. The COVID-19 pandemic has resulted in the publication and dissemination of numerous professional organization resource pages on stress, coping, and related matters (37, 38). However, studies that evaluate both COVID-19-related stress and exposure, as well as coping and biopsychosocial responses, are needed to understand the mental health needs of low socioeconomic status adults in the central NC region. Doing so offers the possibility of developing a tailored intervention program specific to the region's needs.

## The Present Study

Groups that have been economically and socially marginalized in the central NC region may disproportionately experience stress, negative reactions, and psychological disruptions caused by COVID-19. Among low-income adults in the central NC region, the aims of this community-engaged needs assessment were:

**Aim 1**: To quantify COVID-19 specific exposure, stress, and responses.
**Aim 2**: To assess levels of mental health concerns and coping strategies and beliefs.
**Aim 3**: To identify COVID-19 and coping-related risk and protective factors for mental health outcomes.

## Materials and Methods

### Community-Engaged Partnership

This study was conducted with help from a community-academic partnership (39). Psychology for All (40) is a Charlotte area non-profit aimed at reducing barriers to mental health services for people with lower incomes. The university team comprised community-focused researchers in public health, psychology, and social work. Consistent with community-academic partnership principles (39), problem identification was aimed at helping solve pandemic and health-related problems of interest to constituent partners. This project served as an initial needs assessment to inform community action and public health program development.

### Procedure

University investigators obtained Institutional Review Board approval for this assessment. Data were collected in late July of 2020. Convenience sampling was used *via* an online Qualtrics-administered self-report survey constructed for distribution by Psychology for All (40) and its constituent community partners *via* email listserv and social media distribution. Importantly, the following partners all serve ethnic, racial and/or sexual minority groups and people experiencing poverty. We engaged with these partners specifically to reach groups disproportionately experiencing health disparities in Charlotte, NC. Partners sharing the survey opportunity were C4 Counseling, The Harvest Center, Care Ring, Time Out Youth, Westside Education Think Tank, and UNC Charlotte School of Social Work field education partners (41–46). Psychology for All distributed a standard email advertisement of the survey to individual administrative contacts at each constituent partner. Partners shared the email advertisement, which included study goals, participant risks/benefits and the survey link, to their respective clientele. Response rate could not be tabulated because partners did not report back overall listserv sizes.

The informed consent contained information about the study aims, procedure, investigators and contact information, participant rights, and renumeration details. Potential participants indicated consent by selecting “yes” for their willingness to participate in the online consent form. Potential participants first completed the study screener to confirm study eligibility criteria of (1) 18 years of age or older, (2) annual household income of $60,000 or less, and (3) resident in Charlotte-Mecklenburg or surrounding counties (i.e., Gaston, Lincoln, Cabarrus, and Union). The screener survey immediately ended if a potential participant did not meet study inclusion criteria. All subsequent measures (see below) were presented in randomized order, so as to avoid response set effects. Information regarding Psychology for All's online therapy service application, *Psychology Today*'s mental health provider locator, and national crisis phone and text line were given on each survey page and in consent/debriefing documents. Upon survey completion, participants were provided with a written debriefing page. They were also offered the opportunity to provide an email address and preference for a $20.00 Amazon or Walmart e-gift card. The survey took approximately 15–20 min to complete.

### Participants

Online Supplement 1 contains a demographic summary. Participants (*N* = 156) were primarily from Charlotte/Mecklenburg or Lincoln County and were born in the United States. Most participants were either White or Black/African American, between 36 and 55 years of age (*n* = 91; 58.3%), and identified evenly as either men (*n* = 79; 51.0%) or women (*n* = 75; 48.4%). Further, the majority of participants were employed either full or part-time, had an Associate's degree or less, and were insured.

### Measures

#### Demographics

A demographics form first screened participants for age, annual household income, and county of residence, followed by gender, race, ethnicity, insurance status, education level, employment status, and whether the person had been advised to see a mental health provider.

#### COVID-19 Exposure, Stress and Responses

COVID-19 exposure and adjustment measures developed for community research use by the Department of Veterans Affairs were used in the present study. The Coronavirus Stress Survey (47) comprises 10 binary response (i.e., no/yes) questions capturing virus exposure, illness, and difficulties (e.g., medical challenges, familial responsibilities) associated with COVID-19, as well as COVID-19 media exposure. Respondents indicated whether the events happened to themselves and/or someone they know. A total score was tabulated each for personal and other known person stressors. Internal consistency values (Cronbach's α) for the respective COVID-10 stress self (0.54) and other (0.56) values were low. The Coronavirus Response Scale-10 (CRS-10) (48) assesses specific domains of impact such as pain, social support, physical activity, and emotional distress. Items are scored and used individually.

#### Depressive Symptoms

The Patient Health Questionnaire-2 (PHQ-2) (49) is a two-item screening tool used to assess depressed mood and little interest/pleasure in activities. Items are indicated on a 4-point Likert scale ranging from 0 (not at all) to 3 (nearly every day). Items are summed to provide both total and cut scores (>3) to identify those at risk for severe depression. Internal consistency (Cronbach's α) in the present sample was 0.64. As a screening tool for major depressive disorder, the PHQ-2 cut off score demonstrates high sensitivity (83%) and specificity (92%). Additionally, scores on the PHQ-2 are associated with mental health and social and physical functioning (49).

#### Anxiety Symptoms

The Generalized Anxiety Disorder-7 (GAD-7) (50) is a seven-item questionnaire capturing seven domains of anxiety symptoms (e.g., feeling nervous, worrying). Items are indicated on a 4-point Likert scale ranging from 0 (not at all) to 3 (nearly every day). Responses are summed for a total score, which is subsequently classified into four severity categories and a clinical cut-off score (>10). Internal consistency (Cronbach's α) for the total score in the present sample was 0.79. The GAD-7 demonstrates excellent internal consistency (Cronbach's α) among general population samples (α = 0.92) and the sensitivity (89%) and specificity (82%) of the clinical cut-off are high. Scores on the GAD-7 are significantly positively correlated with other anxiety scales, including the Beck Anxiety Inventory (50).

#### Suicide Risk

The Suicidal Ideation Attributes Scale (SIDAS) (51) contains five items assessing suicidal thinking, controllability, and impact. Items are indicated on a variably anchored 11-point Likert scale. Responses are summed to provide a total and cut score (>21) for identification of those at risk for suicide. The SIDAS demonstrates excellent internal consistency among community samples (α = 0.91) and the cut-off score has excellent specificity in identifying individuals at risk of suicidal behavior. Internal consistency (Cronbach's α) in the present sample was 0.92. Additionally, items on the SIDAS are significantly positively associated with other measures of suicidal ideation, such as the Columbia Suicide Severity Scale, as well as measures of depression (PHQ-9) and anxiety (GAD-7) (51).

#### Post-traumatic Stress Disorder (PTSD) Symptoms

The Post-traumatic Checklist-2 (PCL-2) (52–54) is a two-item screener of key post-traumatic symptoms (e.g., intrusive thoughts/images). The 2-item version of the PCL contains the two items from the longer PCL-6 that were most correlated with total score (53). Responses are indicated on a 5-point Likert scale ranging from 1 (not at all) to 5 (extremely). Responses are summed to provide a total score and cut score (>4) for identifying probable PTSD diagnosis. Internal consistency (Cronbach's α) for the total score in the present sample was 0.69. The sensitivity of the PCL-2 cutoff in identifying individuals with PTSD is high (0.97) (52).

#### Problematic Drinking

The Alcohol Use Disorders Identification Test-C (AUDIT-C) (55) is a three-item inventory assessing problematic alcohol use (e.g., frequency of binge drinking). Items are indicated on a 5-point variably anchored Likert scale ranging from 0 to 4. Responses are summed, providing a summed score and cut-score (>4 for men; >3 for women) to identify individuals at risk for alcohol abuse. Internal consistency (Cronbach's α) for the total score in the present sample was 0.52. The AUDIT-C is significantly correlated with other measures of hazardous drinking (56). Further, the AUDIT-C demonstrates acceptable internal consistency (α = 0.70) (57) and is effective in screening for alcohol abuse/dependence (sensitivity = 0.88) (55).

#### Coping and Resilience

The Brief COPE (58) is a 28-item measure containing 14 coping style subscales. We selected three items to assessing venting, active coping, and reframing coping styles. As indicated in the original publishing of the scale, the full instrument does not need to be administered and items and scales can be selected for administration (58). Internal consistency (Cronbach's α) values for Brief COPE subscales used in the present sample were 0.62 for active coping, 0.23 for venting, and 0.59 for positive reframing. The Coping Self-Efficacy Scale (59) was used to assess three areas of beliefs about one's ability to use the following coping strategies: thought stopping, problem-focused coping, and getting social support. Items are indicated on an 11-point Likert scale ranging from 0 (I cannot do this at all) to 10 (I'm certain that I can do this) and summed for a total score. Internal consistency of the CSE subscales is good (thought stopping: α = 0.91, problem-focused coping: α = 0.91, getting social support: α = 0.80). Additionally, CSE subscales are associated with measures of psychological distress (e.g., anxiety, perceived stress), well-being (e.g., optimism), social support, and ways of coping. CSE subscale internal consistency values (Cronbach's α) in the present sample were 0.92 for problem-focused coping, 0.91 for thought stopping, and 0.93 for getting social support. Finally, the Connor-Davidson Resilience Scale−2 item (CD-RISC2) (60) served as a screener for participants' ability to bounce back from difficult circumstances. The CD-RISC2 is significantly negatively correlated with perceived stress and with overall score on the full CD-RISC (60). Internal consistency (Cronbach's α) for the CD-RISC2 total score was 0.73 in the present sample.

Internal consistencies for the current study are reported in Table 1.

**Table 1:** Correlation matrix of COVID-19 related stress and responses, coping factors, and mental health outcomes.

**MH correlate**	**Depression**	**SI**	**PTS**	**Anxiety**	**Alcohol use**	**α**
COVID-19 stress–self total	0.16	−0.08	0.15	0.03	0.01	0.56
COVID-19 stress–other total	**0.32**	**0.37**	**0.21**	**0.30**	**0.46**	0.54
CRS-10 stress response	***0.17***	***−0.21***	***0.26***	0.12	−0.09	-
CRS-10 interaction with friends and family	−0.02	−0.03	−0.08	**−0.24**	−0.02	-
CRS-10 emotional distress response	**0.31**	0.04	***0.21***	***0.17***	0.10	-
CRS-10 physical activity	***0.18***	**0.32**	0.09	***0.18***	***0.18***	***-***
CRS-10 use of alcohol and illicit drugs	**0.37**	**0.36**	**0.35**	**0.35**	**0.42**	**-**
CRS-10 use of prescription medication	***0.21***	**0.43**	***0.22***	***0.23***	***0.26***	***-***
CRS-10 pain	**0.33**	**0.34**	**0.36**	**0.29**	***0.25***	***-***
CRS-10 fear or worry	**0.28**	0.03	**0.43**	**0.36**	0.15	-
CRS-10 effort to cope with stress	***0.19***	0.04	***0.26***	0.14	0.01	-
CRS-10 overall sense of well-being	0.02	0.12	0.12	0.01	0.14	-
Brief resilience	−0.14	***−0.29***	***−0.16***	**−0.29**	***−0.22***	0.73
Active coping	−0.06	**−0.43**	−0.03	−0.11	**−0.29**	0.62
Venting	***0.23***	***0.25***	**0.34**	**0.31**	0.13	0.23
Positive reframing	−0.10	***−0.29***	−0.09	−0.07	−0.15	0.59
Problem-focused coping beliefs	***−0.26***	**−0.40**	−0.13	**−0.33**	***−0.25***	0.92
Thought stopping beliefs	−0.15	***−0.24***	−0.05	***−0.17***	−0.05	0.91
Getting social support beliefs	−0.09	***−0.30***	−0.04	−0.14	−0.08	0.93
α	0.65	0.91	0.69	0.79	0.52	-

*Bold font denotes p < 0.001; Bold italics font p < 0.05*.

*MH, mental health; SI, suicidal ideation; PTS, post-traumatic stress symptoms; Anxiety, generalized anxiety symptoms; COVID-19 Stress, Coronavirus Stress Survey Total Score (Self or Other); CRS-10, Coronavirus Response Scale-10*.

### Data Analysis

Aim 1 and Aim 2 analyses were accomplished using simple frequency counts. Total mental health outcome scores were converted to categories using clinical cut-score values summarized in the measures section. Bivariate correlations were used as the first analytic step in Aim 1 in order to identify bivariate risk and protective factors of mental health outcomes. Following guidelines in the literature (61), multivariate regression was then employed to (a) select demographic covariates of mental health outcomes, and (b) identify the most robust risk and protective factors for mental health outcomes.

## Results

### COVID-19 Specific Exposure, Stress, and Responses

Table 2 contains results from the Coronavirus Stress Survey. Less than one-third of participants reported direct exposure or illness, but almost three-quarters reported knowing someone close who was affected by COVID-19. The most common self-reported coronavirus-related stressors were job exposure, lost job/income, and increased home responsibilities. The most common reported stressors for known close persons were lost job/income, increased home responsibilities, and difficulties getting basic necessities (e.g., medication). A total of 83.4% of the sample reported consuming one or more hours per day of COVID-19 related media information (e.g., TV, Twitter, Facebook).

**Table 2:** Self- and other-experienced COVID-19 related exposure and stressors.

**Coronavirus related experience**	**Happened to me–*n* (%)**	**Happened to someone close to me–*n* (%)**
1. Become ill from possible or certain exposure to the coronavirus	46 (29.5%)	111 (71.2%)
2. Job requires possible exposure to coronavirus	73 (46.8%)	65 (41.7%)
3. Lost job or lost income due to the coronavirus pandemic	72 (46.2%)	84 (53.8%)
4. Increased responsibilities at home due to the coronavirus pandemic	73 (46.8%)	88 (56.4%)
5. Difficulty getting food, medication or other necessities due to the coronavirus pandemic	52 (33.3%)	76 (48.7%)
6. Difficulty getting needed social support due to the coronavirus pandemic	64 (41.0)%	71 (45.5%)
7. Lost health insurance due to the coronavirus pandemic	40 (25.6%)	70 (44.9%)
8. Went on public food assistance due to the coronavirus pandemic	54 (34.6%)	62 (39.7%)

A total of 85 participants provided narrative responses regarding additional COVID-19 related concerns. Responses fell into the following categories (a respondent could provide more than one type): job or financial loss (*n* = 14); decreased socialization or being stuck at home (*n* = 13); personal mental health or negative mood (*n* = 9); job pressures (*n* = 5); no school for kids (*n* = 5); know someone who died of COVID-19 (*n* = 5); having to engage in preventive practices (e.g., wearing a mask) (*n* = 4); and child rearing (*n* = 4). Common responses to the Coronavirus pandemic were cataloged using the CRS-10 items (see Table 1 for items). Descriptive patterns were similar for all 10 items, with average scores falling approximately at the mid-point (*M*_range_=2.66 to 3.24, *SD*_range_=0.96 to 1.16). These scores reflect responses to the pandemic as “about the same” as compared to before the pandemic.

### Mental Health Concerns and Coping-Related Strategies

Table 3 contains descriptive summaries for mental health outcomes and coping-related scales. Scores on PTSD and generalized anxiety screeners indicate elevated levels among this sample. Clinical cut scores derived from source articles (see measures section) show concerning rates of probable risk (in descending order) for post-traumatic stress, depression, problematic drinking, generalized anxiety, and suicide risk. With the exception of venting, participants reported scale midpoint levels of all coping-related factors. Venting was used less than the midpoint.

**Table 3:** Mental health and coping-related descriptive statistics.

**Mental health or coping related factor**	***M* (*SD*)**	**Mean score label**	***n* (%) above cut score suggesting increased risk**	**Interpretation of elevated risk**
Depression	2.47 (1.46)	No risk	83 (52.2%)	Probable risk for depression
Suicidal thinking	14.62 (11.50)	No risk	63 (40.4%)	Elevated suicide risk
Post-traumatic stress	5.61 (1.82)	Possible PTSD	130 (83.3%)	Possible PTSD
Anxiety	8.09 (4.02)	Moderate anxiety	67 (43.0%)	Moderate or worse
Alcohol use - men^a^	3.39 (1.50)	No risk	40 (50.6%)	Problematic drinking
Alcohol use – women	2.67 (1.91)	No risk	38 (50.0%)	Problematic drinking
Resilience	3.38 (0.88)	Neutral	-	-
Active coping	2.69 (0.70)	Doing a medium amount	-	-
Venting	2.49 (0.70)	Doing a little bit	-	-
Positive reframing	2.77 (0.75)	Doing a medium amount	-	-
Problem-focused coping beliefs	5.24 (2.07)	Moderately certain I can do this	-	-
Thought stopping beliefs	5.26 (2.18)	Moderately certain I can do this	-	-
Getting social support beliefs	5.49 (2.26)	Moderately certain I can do this	-	-

*M, mean; SD, standard deviation; PTSD, Post-traumatic stress disorder*.

a *AUDIT-C requires breakdown by gender for use of cut-scores*.

### COVID-19 and Coping-Related Risk and Protective Factors for Mental Health Outcomes

Table 1 contains a bivariate correlation matrix of the following factors related to mental health outcomes: (1) all summed COVID-19 stress survey scores for self and other (both derived from items on Table 2, score range 0–8); (2) HCRS-10 items; and (3) coping-related factors. Correlates most robustly related to better mental health outcomes included resilience and problem-focused coping beliefs. The strongest correlates of negative mental health outcomes were COVID-19 related stress others (Coronavirus Stress Survey–others subscale); responses to the pandemic–physical activity level, pain, alcohol/drug use, prescription medication use, fear/worry (CRS-10 items); and the coping domain of venting.

Prior to evaluating bivariate correlates of mental health outcomes, we sought to identify necessary demographic covariates. Due to low cell sizes in many categories, demographics were recoded for regression analyses: gender (1 = male, 2 = female [1 ‘other' dropped']), age (1 = 18–35, 2 = 36+), county (1 = Charlotte/Mecklenburg [the inner lying urban county], 2 = other), race (1 = White, 2 = racial minority), ethnicity (1 = Non-Hispanic; 2 = Hispanic or other minority), education (1 = High school/GED or less, 2 = Associate's Degree or higher), and employment status (1 = employed, 2 = unemployed or retired). Binary demographic variables were entered into a multivariate regression model with all mental health outcomes included. Demographics with significant overall multivariate effects on mental health outcomes were retained as control variables for further analysis. Only gender (Wilks' λ=0.86, F[5, 132] = 4.45, *p* = 0.001), county (Wilks' λ=0.91, F[5, 132] = 2.70, *p* = 0.02), and employment status (Wilks' λ = 0.89, F[5, 132] = 3.16, *p* = 0.01) were retained for further analyses.

A multivariate regression model was constructed to identify the most robust risk and protective factors of mental health outcomes. The model featured outcomes of depression, suicidal ideation, post-traumatic stress, generalized anxiety, and alcohol use (*r*s range = 0.30 to 0.61, *p*s < 0.001). COVID-19 stress and response as well as coping-related factors displaying significant correlations with two or more mental health outcomes (see Table 1) were included as main effect predictors in the multivariate model. Gender, county, and employment status were demographic covariates. This resulted in a final set of regression model predictors of: gender, employment status, county, COVID-19 Stress (other), stress response, emotional distress, physical activity, use of alcohol/drugs, use of prescription medication, pain, fear/worry, effort to cope with stress, brief resilience, active coping, venting, problem-focused coping beliefs, and thought stopping beliefs. Table 4 contains multivariate test statistics for each predictor; only significant multivariate predictors were inspected as univariate risk or protective factors. Significant multivariate predictors (moderate-to-large effects) were employment status, COVD-19 related Stress (other), fear/worry reaction to the pandemic, active coping, venting, and problem-focused coping beliefs. Significant risk and protective factors by mental health outcome were as follows:

1. Depression model: COVID-19 Stress (other) (*B* = 0.29, se*B* = 0.12, *p* = 0.01); venting (*B* = 0.23, se*B* = 0.11, *p* = 0.04); problem-focused coping beliefs (*B* = −0.53, se*B* = 0.19, *p* = 0.007).
2. Suicidal ideation model: COVID-19 Stress (other) (*B* = 1.69, se*B* = 0.79, *p* = 0.03); active coping (*B* = −2.69, se*B* = 0.82, *p* = 0.001); venting (*B* = 2.84, se*B* = 0.77, *p* < 0.001).
3. Post-traumatic stress model: Fear/worry response (*B* = 0.58, se*B* = 0.16, *p* < 0.001); venting (*B* = 0.49, se*B* = 0.14, *p* = 0.001).
4. Generalized anxiety model: Employed (*B* = 1.84, se*B* = 0.76, *p* = 0.02); COVID-19 stress (other) (*B* = 0.67, se*B* = 0.30, *p* = 0.02); fear/worry response (*B* = 1.41, se*B* = 0.33, *p* < 0.001); problem-focused coping beliefs (*B* =−1.42, se*B* = 0.49, *p* = 0.005).
5. Alcohol use model: COVID-19 stress (other) (*B* = 0.53, se*B* = 0.13, *p* < 0.001).

**Table 4:** Multivariate^*^ regression model statistics predicting mental health outcomes.

**Predictor**	**Wilks' λ**	**F (df)**	***p*-value**	** ${\text{η}}_{\text{p}}^{2}$ **
**Intercept**	**0.09**	**270.30 (5, 127)**	**<0.001**	**0.91**
Gender	0.92	2.28 (5, 127)	0.05	0.08
County	0.99	0.40 (5, 127)	0.85	0.01
**Employment status**	**0.88**	**3.33 (5, 127)**	**0.008**	**0.11**
**COVID-19 stress (other)**	**0.82**	**5.59 (5, 127)**	**<0.001**	**0.18**
HCRS-10 Stress Response	0.92	2.13 (5, 127)	0.07	0.08
HCRS-10 Emotional Distress	0.93	1.77 (5 127)	0.12	0.06
HCRS-10 Physical Activity	0.98	0.57 (5, 127)	0.72	0.02
HCRS-10 Use of Alcohol/Drugs	0.93	2.01 (5, 127)	0.08	0.07
HCRS-10 Use of Prescription Medication	0.94	1.71 (5, 127)	0.14	0.06
HCRS-10 Pain	0.97	0.72 (5, 127)	0.61	0.03
**HCRS-10 fear/Worry**	**0.83**	**5.15 (5, 127)**	**<0.001**	**0.17**
HCRS-10 Effort to Cope with Stress	0.98	0.54 (5, 127)	0.75	0.02
Resilience	0.97	0.78 (5, 127)	0.57	0.03
**Active coping**	**0.89**	**3.17 (5, 127)**	**0.01**	**0.11**
**Venting**	**0.83**	**5.01 (5, 127)**	**<0.001**	**0.16**
**Problem-Focused coping beliefs**	**0.91**	**2.51 (5, 127)**	**0.03**	**0.09**
Thought Stopping Beliefs	0.98	0.48 (5, 127)	0.79	0.02

*Bold font denotes significant multivariate predictor*.

*HCRS-10, Hilgeman Coronavirus Response Scale-10*.

* *Multivariate analyses allow for inclusion of multiple dependent variables in one model and provide overall omnibus tests for each predictor^61^*.

*As such, statistics in the table are for the overall multivariate effect on the collection of mental health outcomes. Outcome specific model effects were as follows: Depression model: F(17, 131) = 4.60, p <0.001, Adj. R^2^ = 0.29; Suicidal ideation model: F(17, 131) = 8.86, p <0.001, Adj. R^2^ = 0.47; Post-traumatic stress model: F(17, 131) = 4.85, p <0.001, Adj. R^2^ = 0.31; Generalized anxiety model: F(17, 131) = 6.88, p <0.001, Adj. R^2^ = 0.40; Alcohol use model: F(17, 131) = 5.57, p <0.001, Adj. R^2^ = 0.34*.

## Discussion

The current study was developed in response to the global call to action of the COVID-19 suicide prevention research collaboration (4). The present study contributes to that call by gaining a picture of stress and mental health among low-income adults in central NC. First, we examined the prevalence of clinically elevated mental health and suicide risk scores at the height of the pandemic. Next, we assessed how COVID-19 related exposure and responses were associated with mental health and suicide outcomes among adults with lower incomes. Findings from the present study indicate increased needs for mental health care and services in the region. We observed elevated rates of probable risk or need for further evaluation for post-traumatic stress, depression, problematic drinking, generalized anxiety, and suicide. The rates of mental health outcomes in the current sample were elevated compared to pre-pandemic global prevalence rates (5). Additionally, the rates of mental health challenges observed within our sample of NC adults are higher than national trends in the CDC report at a similar point in time (36). While measurement differences between the CDC report and our study may explicate varying mental health and substance use rates, it is also possible that the individuals in our low-income NC sample face additional layers of stress that amplify mental health and substance use outcomes. However, given that our rates were obtained *via* use of very brief screening instruments, further evaluation from licensed healthcare providers would be necessary for formal diagnosis.

A variety of COVID-19 stressors and problematic responses were observed. We observed high rates of COVID-19 illness and exposure. Common stresses resulting from the COVID-19 pandemic included job/financial loss, increased home responsibilities, and difficulties with basic necessities (e.g., getting medication). Such stressors ranged from 26 to 47% of the sample, with higher rates of some stressors among other known persons (see Table 2). Open-ended responses illuminated additional impacts such as decreased socialization and negative emotions. Themes of pandemic-driven financial stress, socialization difficulties, fear of illness, and concerns about mental health are consistent with prior literature (18, 21–23). Finally, the fact that CRS-10 items were positively correlated with many mental health concerns suggests that stress responses may reflect ineffective coping strategies (e.g., physical activity) or additional symptoms of stress reactions to the pandemic (e.g., pain).

The third study aim concerned identifying pandemic-related risk factors for reduced mental health outcomes. Extending existing literature highlighting the overall severity of fear and worry during the pandemic (11, 62), COVID-19 related fear/worry was also a prominent risk factor for post-traumatic stress and anxiety symptoms in particular. Further, our correlational and regression findings suggest that knowing someone else struggling with COVID-19 related stress was more problematic for personal mental health than one's own exposure or stress. This fact is pivotal to understanding the regional effects of the pandemic. Other salient mental health risk factors included a venting coping style (for both symptoms of depression and post-traumatic stress). Consistent with a few national studies (23, 24), active coping and problem-focused coping beliefs show some promise as protective factors for depression and suicide risk, respectively, during the pandemic.

### Limitations

This study possesses several clear limitations that temper conclusions and recommendations. Convenience sampling *via* online methods placed an obvious limitation on who could be reached for the needs assessment. Further, the sample was intentionally restricted by certain demographics, and the racial composition was unclear due to a survey readability concern (see Online Supplement Note). Additionally, the sample size of the current study was small. Moreover, we could not calculate a survey response rate due to absence of the total possible population reached with the study advertisement. Thus, generalizability to the full region is quite restricted. Also, as is often the case with intentionally brief clinical screening instruments (e.g., PHQ-2, COVID-19 stress, Brief COPE screeners), internal consistency was low for several measures. Psychometric research suggests that a low number of items (four or less) can cause low internal consistency. Low reliability offers a potential explanation of any non-significant findings in the present study because low internal consistency limits the ability to detect correlations with other measures. This limitation does not, however, render the screening tools useless in applied/field public health research. The benefit of short screeners is real world efficiency in identifying persons who may be at risk for a number of clinical conditions or stress coping problems, and to evaluate those relationships among the persons who need assistance. Future research, program evaluation, and follow-up in this area should employ longer clinically-relevant mental health and coping tools. Finally, the online data collection administration may have limited survey access; although many people in urban areas are connected to the Internet, prioritizing populations who may experience financial and technological resource deficits compromised our ability to reach the full scope of community partners. Although we employed a community-engaged strategy, further community partnerships with local faith and other community leaders and agencies can expand additional needs assessments and follow-up public health programming.

### Public Health and Research Implications

Findings from this study suggest possible avenues for a regional public health strategy to address the adverse mental health effects of the pandemic in the Charlotte, NC region. A public health approach should include the following facets. First, given the high positive screening rates for mental health problems, programs should focus on regional investment in virtual training of mental health providers in leading assessment practices and evidence-based therapies to treat mental health and substance use disorders. Such therapeutic approaches may include the Collaborative Assessment and Management of Suicide (CAMS) (63), Dialectical Behavior Therapy (DBT) (64), and Cognitive-Behavioral Therapy (CBT) techniques such as Motivational Interviewing and Cognitive Restructuring (65). Given that access to specialty mental health care is limited, particularly for individuals with low incomes, embedding mental health providers in medical settings where COVID-19 diagnostic procedures and heightened stress may be present (e.g., primary care, community health clinics; emergency departments) is critical to reaching those in need of services.

Research also demonstrates efficacy of online alcohol interventions, especially when accompanied by therapeutic principles and person support (66). In light of logistical limitations (e.g., social contact, limited transportation) the pandemic may impose additional impediments on people with lower incomes; therefore, more equitable efforts in the region may promote existing virtual alcohol interventions such as virtual 12-step programs or cognitive-behaviorally based therapies. Selection and implementation of online alcohol interventions should occur in consultation with a qualified substance use expert. Alternatively, mental health service providers may seek to implement online therapy groups for persons with problematic substance use in the region; such programs can tackle specific problems identified in this needs assessment.

A variety of community-based responses may further address the stress and mental health implications of the pandemic. For instance, community-based screenings are a common practice to detect persons at risk for various mental health and alcohol use disorders (67). We recommend the widespread use of COVID-19 stress and general mental health screening instruments such as those employed in this assessment. They may be implemented in-person or online by partnering with regional agencies, emergency department or primary care facilities, or other non-profit entities. When used online, screening tools should be accompanied by clear instructions on how to reach a provider. Another community-focused strategy is the design of a comprehensive pandemic awareness campaign. Social media, radio, newspaper print, podcast, and other platforms can unify public health messaging such as the importance of remaining socially distanced yet connected, mental health warning signs and benefits of therapy, and free, brief coping skills tools. Additionally, awareness campaign design should employ principles of behavior change theories such as the Theory of Planned Behavior (68). Campaign messages can be augmented by public health educational materials for social media and print distribution *via* non-profit, academic, and healthcare entities in the region.

Our findings also have implications informing public health research moving forward. As COVID-19 becomes endemic, future areas of study will need to identify suitable assessment tools including standardized domains of data collection to monitor the mental health consequences of adjusting to outbreaks and public health interventions to address COVID-19 over time. We utilized two unpublished inventories developed: The Coronavirus Stress Survey (CSS) and Coronavirus Response Scale (CRS). We selected these tools as they are consistent with large-scale research efforts by a leading national healthcare agency, namely the Department of Veterans Affairs. Our findings provide preliminary data suggesting these tools may be useful for future COVID-19 stress and related research. However, other relevant emerging measures exist, such as the COVID-19 Anxiety Scale, (69) that also warrant further study. Mixed-methods findings from our study highlight a number of possible outcomes for COVID-19 stress, coping, and mental health intervention, and program evaluation research. Namely, COVID-19 stress, coping self-efficacy, and a myriad of mental health domains may be the subject of intervention development and evaluation moving forward.

## Data Availability Statement

A de-identified dataset is available by direct contact of the corresponding author at rcramer4@uncc.edu.

## Ethics Statement

The current study involved human participants and was reviewed and approved by UNC Charlotte Institutional Review Board. Participants provided informed consent to participate in this study electronically.

## Author Contributions

AK, RC, CM, AP, MZ, and JL-R: writing. Y-JH, GG, SS, CW, AK, RC, and AM: data collection. RC, AK, and CW: data analysis. Y-JH, RC, AM, and JL-R: getting funding. RC, MZ, and JL-R: supervision. All authors: editing.

## Funding

This work was supported by a generous grant from the Cardinal Innovations Healthcare COVID-19 Relief Fund.

## Conflict of Interest

The authors declare that the research was conducted in the absence of any commercial or financial relationships that could be construed as a potential conflict of interest.

## Publisher's Note

All claims expressed in this article are solely those of the authors and do not necessarily represent those of their affiliated organizations, or those of the publisher, the editors and the reviewers. Any product that may be evaluated in this article, or claim that may be made by its manufacturer, is not guaranteed or endorsed by the publisher.
