# Supplementary material for: COVID-19 Exposure, Stress, and Mental Health Outcomes: Results From a Needs Assessment Among Low Income Adults in Central North Carolina
Source: Front Psychiatry. 2022 Jan 20;12:790468. doi: 10.3389/fpsyt.2021.790468 (PMC8812254; doi:10.3389/fpsyt.2021.790468)
Supplement: Supplementary file 1 [file Table_1.pdf]

***Online Supplement Table 1. Sample Demographic Information*.**

Demographic Characteristic	*n* (%)
Age	
18-25	17 (10.9%)
26-35	42 (26.9%)
36-45	74 (47.4%)
46-55	17 (10.9%)
56-64	5 (3.2%)
64-74	1 (0.6%)
County of Residence	
Charlotte/Mecklenburg	72 (46.2%)
Lincoln	49 (31.4%)
Cabarrus	19 (12.2%)
Gaston	15 (9.6%)
Union	1 (0.6%)
Race	
White	53 (34.0%)
American Indian/Alaskan Native/Other[1]	47 (30.1%)
Black/African American	45 (28.8%)
Asian/Pacific Islander	6 (3.8%)
Multiracial	3 (1.9%)
Biracial	2 (1.3%)
Ethnicity	
Hispanic/Latino(a)	71 (46.7%)
Non-Hispanic/Latino(a)	68 (44.7%)
Other	13 (8.6%)
Health Insurance	
Yes	128 (86.5%)
No	20 (13.5%)

Born in the United States	
Yes	151 (99.3%)
No	1 (0.7%)
Gender	
Man	79 (51.0%)
Woman	75 (48.4%)
Transgender man or woman	1 (0.6%)
Education Level	
Less than high school	3 (1.9%)
Some high school	7 (4.5%)
High school diploma/GED	28 (18.2%)
Associate's degree	85 (55.2%)
Bachelor's degree	25 (16.2%)
Graduate degree	6 (3.9%)
Employment Status	
Full-time	69 (44.2%)
Part-time	51 (32.7%)
Unemployed	26 (16.7%)
More than one job	4 (2.6%)
Retired	1 (0.6%)

[1]The Charlotte-Mecklenburg region comprises less than 0.5% American Indian/Alaskan Native (AI/AN) persons. We investigated possible explanations of random responding or a systematic influence on race responses. AI/AN was the first option presented to respondents and persons indicating this race category were also systematically more likely to report lower education. Thus, we interpret the high rate of AI/AN persons in the dataset as a result of reading comprehension. It is likely that respondents read this as "American" and checked the response. Therefore, race data should be interpreted with caution.
